# Editorial: Design and synthesis of nanocarriers for enhancement of antigen immunogenicity

**DOI:** 10.3389/fbioe.2023.1252844

**Published:** 2023-07-11

**Authors:** Shujuan Yu, Chao Pan

**Affiliations:** State Key Laboratory of Pathogen and Biosecurity, Beijing Institute of Biotechnology, Beijing, China

**Keywords:** nanocarrier, vaccine delivery, immunogenicity, immune enhancement, conjugate vaccine

Since the emergence of COVID-19 in 2019, vaccines have been increasingly recognized as essential in preventing the spread of pandemics. Generally, the effectiveness of a vaccine is mainly determined by three primary factors—antigen, adjuvant, and formulation. Antigen could trigger specific immune responses, adjuvant could amplify the immune reactions, while formulation determines vaccine’s uptake by antigen-presenting cells and activation of specific T cells. Of these, antigen is the most critical element of vaccine design. Recent advances in vaccine research have focused on developing subunit vaccines which have more distinct components and higher levels of safety than inactivated and attenuated ones. These subunit vaccines contain individual protein or polysaccharide antigens, even smaller epitope peptides with low immunogenicity. To improve the immune response, researchers have found that coupling the antigen with an appropriate carrier, such as a nanocarrier, has shown potential in enhancing antigen immunogenicity. Nanocarriers, due to their unique lymph node drainage and immune activation abilities, have attracted more attention for vaccine design. There are currently various types of nanocarriers available, including toxin proteins like cholera toxin B subunit (CTB), virus-like particles (VLP), outer membrane vesicles (OMV), self-assembled particles, liposomes, emulsions, and polymeric particles, that provide potential solutions to explore the effectiveness of nanocarriers in vaccine preparation.

Conjugated vaccine is a typical example of enhancing antigen immunogenicity through an appropriate vector. Different from the currently marketed conjugate vaccines, which are chemically synthesized and use protein monomer as carrier, Li et al. utilized pentamer CTB as carrier protein to produce a *B. abortus* conjugate vaccine with the size of about 10 nm. It is interesting that in this design, the conjugation of polysaccharide antigens and proteins was directly synthesized in *E. coli*, avoiding the complex steps of chemical methods. This bio-method is becoming a new trend in development of conjugate vaccines. Their results showed that the conjugate vaccine was successful in eliciting humoral immune responses and producing specific antibodies that protected mice against *B. abortus* A19 in challenge experiments. Additionally, Sun et al. developed an OMV-based click vaccine through the SpyCatcher-SpyTag system. In the results, the vaccine elicited strong humoral and cellular immune responses, that were greater than those produced by the alum-adjuvanted vaccine. This click vaccine also protected mice from lethal challenges by *S. aureus* Newman, indicating its potential to combat emerging clinical isolates. Finally, Gao et al. developed a pertussis vaccine that includes oligosaccharides conjugates and pertussis toxin, which was successful in initiating a cell immune response and significantly decreasing lung bacterial loads of *B. pertussis* in a mouse aerosol infection model. These above studies highlight the potential of bioconjugate vaccines, OMV-based click vaccines, and two-component vaccines in providing potent immunogenicity against infectious diseases, indicating that for different types of antigens, whether polysaccharide or protein antigens, the introduction of appropriate carriers, especially nanocarriers, can greatly improve the specific immune responses.

Subunit vaccines often lack the potency needed to create strong and long-lasting protective immune responses against harmful pathogens. Nanoparticle-based delivery systems offer a promising approach to overcome limitations of traditional vaccine adjuvants (Das and Ali, 2021). Some benefits of these nanovaccines include targeted delivery, enhanced antigen presentation, stimulation of the innate immune system, and a strong T cell response, while remaining safe for use in combatting infectious diseases and cancers. In the treatment of chronic bacterial prostatitis, free drugs, such as antibiotics, have limited efficacy due to difficulty penetrating the prostate epithelium and targeting inflammatory tissues. Nanotechnology presents an opportunity to address this issue by developing targeted nanoparticle delivery strategies, relying on specific nanoparticle physicochemical properties. Hu et al. found that folic acid-modified nanoparticles with specific particle size and administration method had optimal targeting efficiency to inflammatory prostate tissues of chronic bacterial prostatitis. Additionally, Zhao et al. demonstrated a new nanoengineering approach that improves the photoacoustic response from biocompatible materials, incorporating optical absorbers into the shell of gaseous nanobubbles to enhance their signal generation. This study shows that nanoengineering approaches have the potential to enhance the specificity of targeting and molecular imaging in pre-clinical cancer models.

Microneedles have gained popularity in the field of vaccine delivery due to their ease of use, minimal pain, and convenience (Sheng et al., 2021). Additionally, they show great potential as a less-invasive transdermal drug delivery route, which has generated significant interest. Microneedles offer various advantages, including nonselective loading capacity, simple operation, and good biocompatibility (Zhang et al., 2023). Direct delivery of therapeutics to the skin makes microneedles a valuable tool, particularly in the treatment of cutaneous diseases, such as psoriasis, atopic dermatitis, skin and soft tissue infections, superficial tumors, axillary hyperhidrosis, and plantar warts. Chen et al. describe different techniques for fabricating microneedles and the application of these techniques in the treatment of various cutaneous diseases. This article further explores the potential of microneedle-mediated drug delivery for dermatological disease treatment.

Developing effective vaccines requires enhancing immunogenicity, which can be achieved through various strategies such as selecting better antigens, using novel adjuvants and immune-enhancing agents, developing multi-valent vaccines, and improving formulation design. Recently, nanoparticle technology has emerged as a promising solution for vaccine development. Nanoparticles can act as vaccine carriers, enhancing immunogenicity and increasing targeting by using specific peptides. Additionally, their size allows for adjuvant effects and the incorporation of multiple antigens, making them ideal candidates for vaccine delivery. An ideal nanovaccine design should target antigen-presenting cells and enable antigens delivery across biological barriers to reach target organs. In addition, advancements in computational modeling and data analysis are helping to accelerate the design of new nanoparticle-based vaccines, contributing to their development as the next-generation of vaccines.
